# Dietary and supplemental intake of vitamins C and E is associated with altered DNA methylation in an epigenome-wide association study meta-analysis

**DOI:** 10.1080/15592294.2023.2211361

**Published:** 2023-05-26

**Authors:** Amena Keshawarz, Roby Joehanes, Jiantao Ma, Gha Young Lee, Ricardo Costeira, Pei-Chien Tsai, Olatz M. Masachs, Jordana T. Bell, Rory Wilson, Barbara Thorand, Juliane Winkelmann, Annette Peters, Jakob Linseisen, Melanie Waldenberger, Terho Lehtimäki, Pashupati P. Mishra, Mika Kähönen, Olli Raitakari, Mika Helminen, Carol A. Wang, Phillip E. Melton, Rae-Chi Huang, Craig E. Pennell, Therese A. O’Sullivan, Carolina Ochoa-Rosales, Trudy Voortman, Joyce B.J. van Meurs, Kristin L. Young, Misa Graff, Yujie Wang, Douglas P. Kiel, Caren E. Smith, Paul F. Jacques, Daniel Levy

**Affiliations:** a Framingham Heart Study, Framingham, Framingham, MA, USA; b Population Sciences Branch, Division of Intramural Research, National Heart, Lung, and Blood Institute, National Institutes of Health, Bethesda, MD, USA; c Friedman School of Nutrition Science and Policy, Tufts University, Boston, MA, USA; d Department of Twin Research and Genetic Epidemiology, King's College London, London, UK; e Department of Biomedical Sciences, Chang Gung University, Taoyuan, Taiwan; f Genomic Medicine Research Core Laboratory, Chang Gung Memorial Hospital, Linkou, Taiwan; g Research Unit of Molecular Epidemiology, Institute of Epidemiology, Helmholtz Zentrum München, Ingolstädter Landstrasse 1, Neuherberg, Germany; h Institute of Epidemiology, Helmholtz Zentrum München, German Research Center for Environmental Health, München, Germany; i German Center for Diabetes Research (DZD), München-Neuherberg, Germany; j Institute of Neurogenomics, Helmholtz Zentrum München, German Research Center for Environmental Health (GmbH), Neuherberg, Germany; k Institute of Human Genetics, School of Medicine, Technical University of Munich, Munich, Germany; l Chair of Neurogenetics, School of Medicine, Technical University of Munich, Munich, Germany; m Munich Cluster for Systems Neurology (SyNergy), Munich, Germany; n Chair of Epidemiology, Medical Faculty, Institute for Medical Information Processing, Biometry and Epidemiology, Ludwig-Maximilians-Universität München, Munich, Germany; o German Center for Cardiovascular Research (DZHK), München Heart Alliance, Munich, Germany; p Chair of Epidemiology, University Augsburg at University Hospital Augsburg, Augsburg, Germany; q Department of Clinical Chemistry, Faculty of Medicine and Health Technology, Tampere University, Tampere, Finland; r Finnish Cardiovascular Research Center Tampere, Faculty of Medicine and Health Technology, Tampere University, Tampere, Finland; s Department of Clinical Chemistry, Fimlab Laboratories, Tampere, Finland; t Department of Clinical Physiology, Tampere University Hospital, Tampere, Finland; u Research Centre of Applied and Preventive Cardiovascular Medicine, University of Turku, Turku, Finland; v Department of Clinical Physiology and Nuclear Medicine, Turku University Hospital, Turku, Finland; w Centre for Population Health Research, University of Turku and Turku University Hospital, Turku, Finland; x Tays Research Services, Tampere University Hospital, Tampere, Finland; y Faculty of Social Sciences, Health Sciences, Tampere University, Tampere, Finland; z School of Medicine and Public Health, College of Health, Medicine and Wellbeing, The University of Newcastle, Newcastle, New South Wales, Australia; aa Hunter Medical Research Institute, Newcastle, New South Wales, Australia; bb Menzies Institute for Medical Research, University of Tasmania, Hobart, Tasmania, Australia; cc School of Population and Global Health, University of Western Australia, Perth, Australia; dd Nutrition & Health Innovation Research Institute, Edith Cowan University, Perth, Australia; ee School of Medical and Health Sciences, Edith Cowan University, Joondalup, Australia; ff Department of Epidemiology, Erasmus MC University Medical Center, Rotterdam, the Netherlands; gg Centro de Vida Saludable, Universidad de Concepción, Concepción, Chile; hh Department of Internal Medicine, Erasmus MC University Medical Center, Rotterdam, the Netherlands; ii Department of Epidemiology, Gillings School of Global Public Health, University of North Carolina, Hebrew Senior Life, Chapel Hill, North Carolina, USA; jj Department of Medicine, Beth Israel Deaconess Medical Center, Hinda and Arthur Marcus Institute for Aging Research, Hebrew SeniorLife, Boston, MA, USA; kk Department of Medicine, Beth Israel Deaconess Medical Center and Harvard Medical School, Boston, MA, USA; ll Jean Mayer USDA Human Nutrition Research Center on Aging, Tufts University, Boston, MA, USA

**Keywords:** Epigenetics, Vitamin C, Vitamin E, diet, epidemiology

## Abstract

Background: Dietary intake of antioxidants such as vitamins C and E protect against oxidative stress, and may also be associated with altered DNA methylation patterns.

Methods: We meta-analysed epigenome-wide association study (EWAS) results from 11,866 participants across eight population-based cohorts to evaluate the association between self-reported dietary and supplemental intake of vitamins C and E with DNA methylation. EWAS were adjusted for age, sex, BMI, caloric intake, blood cell type proportion, smoking status, alcohol consumption, and technical covariates. Significant results of the meta-analysis were subsequently evaluated in gene set enrichment analysis (GSEA) and expression quantitative trait methylation (eQTM) analysis.

Results: In meta-analysis, methylation at 4,656 CpG sites was significantly associated with vitamin C intake at FDR ≤ 0.05. The most significant CpG sites associated with vitamin C (at FDR ≤ 0.01) were enriched for pathways associated with systems development and cell signalling in GSEA, and were associated with downstream expression of genes enriched in the immune response in eQTM analysis. Furthermore, methylation at 160 CpG sites was significantly associated with vitamin E intake at FDR ≤ 0.05, but GSEA and eQTM analysis of the top most significant CpG sites associated with vitamin E did not identify significant enrichment of any biological pathways investigated.

Conclusions: We identified significant associations of many CpG sites with vitamin C and E intake, and our results suggest that vitamin C intake may be associated with systems development and the immune response.

## Introduction

Methylation of DNA cytosine-phosphate-guanine (CpG) sites reflects a reversible epigenetic modification associated with altered regulation of gene expression. DNA methylation is frequently associated with activation or inactivation of genes and biological pathways, and differential methylation of specific CpG loci has been associated with multiple clinical diseases, including cancer [1–3], diabetes mellitus [4,5], and cardiovascular disease [6]. Oxidative stress, characterized by the production of reactive oxygen species (ROS) that can damage DNA, proteins, and lipids, is one mechanism by which perturbations to DNA methylation patterns can contribute to clinical disease [7–10]. Oxidative stress may upregulate the expression of DNA methyltransferases (DNMTs), which catalyse DNA methylation. Minimizing oxidative stress may help to prevent deleterious DNA methylation patterns and clinical disease.

Dietary intake of antioxidants may provide some protection against oxidative stress [11–14], and may also affect DNA methylation patterns. Vitamin C, or ascorbic acid, is a water-soluble antioxidant that prevents oxidative stress through the reduction of ROS, thereby inhibiting peroxidative processes that can damage cells, DNA, and proteins [15]. Vitamin C is found in the cellular cytosol and extracellular fluid, and it coactivates ten-eleven translocation (TET) enzymes that mediate DNA demethylation via conversion of 5-methylcytosine to 5-hydroxymethylcytosine [16–19]. Vitamin C works synergistically with the lipid-soluble vitamin E [20], or α-tocopherol, another antioxidant that interferes with the propagation of lipid radicals, by reducing oxidized vitamin E and thereby allowing it to continue acting as an antioxidant [21–23].

We sought to evaluate epigenomic patterns associated with dietary intake of vitamins C and E in several large cohorts.

## Methods

### Study design and population

**Figure 1.**
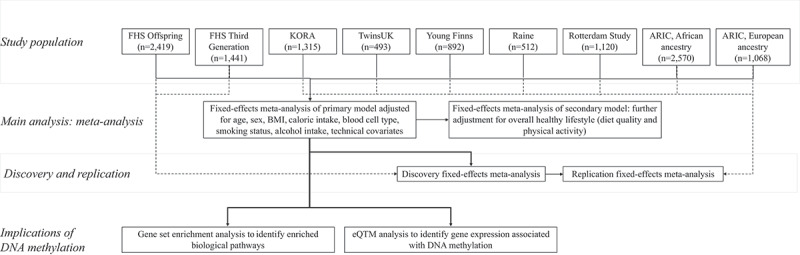
Summary of the overall study design.

The overall study design is presented in Figure 1. Cross-sectional data on DNA methylation and vitamin intake from participants enrolled in eight population-based cohorts were used in this study: the Framingham Heart Study (FHS) Offspring cohort (*n* = 2,419) [24], the FHS Third-Generation cohort (*n* = 1,441) [25], the UK Adult Twin Registry (TwinsUK; *n* = 493) [26], the Kooperative Gesundheitsforschung in der Region Augsburg (KORA) study (*n* = 1,392) [27,28], the Young Finns study (*n* = 892) [29], Generation 2 of the Australia-based Raine Study (*n* = 512) [30], the Rotterdam Study (*n* = 1,120) [31], and the Atherosclerotic Risk in Communities (ARIC) study (*n* = 2,570 with African ancestry, *n* = 1,068 with European ancestry) [32]. All cohorts included participants with European ancestry; the ARIC study additionally included participants with African ancestry. Institutional review committees of each cohort approved this study, and all participants provided written informed consent. Data and analytical codes that support our findings are available from the corresponding author upon request. Detailed cohort descriptions for each of these cohorts are presented in the **Supplemental Materials**.

### Clinical and behavioural risk factors

Clinical and behavioural risk factors included as covariates in statistical models were collected according to each cohort’s specific protocols, as detailed in the **Supplemental Materials**. Body mass index (BMI) was calculated as the ratio of the participant’s measured weight (in kg) to height (in m) squared. Smoking status was obtained based on self-reported data, and participants were categorized as current, former, and never smokers. Self-reported physical activity data were available for the FHS Offspring study, FHS Third-Generation study, YFS, Rotterdam study, and ARIC study and are described in detail in the **Supplemental Materials.**

### Vitamin intake

Dietary and supplementary intake of vitamin C, dietary intake of vitamin E, and overall caloric intake were calculated based on self-reported food frequency questionnaire (FFQ) data or a combination of repeated 24-hour recalls and a FFQ (KORA FF4). The FHS cohorts used the 126-item Harvard-Willett FFQ [33]; the TwinsUK cohort used the 131-item European Prospective Investigation into Cancer and Nutrition (EPIC)-Norfolk FFQ [34]; the KORA cohort used two 24-hour recalls and the Bavarian Food Consumption Survey [35]; the Young Finns Study cohort used a 131-item FFQ developed by the Finnish National Institute for Health and Welfare [36]; the Raine study used a 212-item FFQ developed by the Commonwealth Scientific and Industrial Research Organisation [37]; the Rotterdam Study used a 389-item Dutch FFQ [38]; and the ARIC cohorts used a 66-item modified Willett FFQ [33,39]. Additional details about the study-specific FFQs are described in the **Supplementary Materials.**

Continuous vitamin C intake based on dietary and supplemental intake was categorized into low, medium, and high intake based on sex-specific thresholds for adults recommended by the Institute of Medicine of the National Academies **(Supplemental Table S1)** [40]. Low vitamin C intake was categorized as intake not meeting the recommended dietary allowance; medium vitamin C intake was categorized as intake meeting the recommended dietary allowance up to 300 mg in both men and women; and high vitamin C intake was categorized as any intake above 300 mg in both men and women.

Continuous vitamin E intake based on dietary and supplemental intake was similarly categorized into low, medium, and high intake based on the recommended dietary allowance for vitamin E **(Supplemental Table S1)** [40]. Low vitamin E intake was categorized as intake not meeting the recommended dietary allowance, medium vitamin E intake was categorized as intake meeting the recommended dietary allowance of 15 mg up to 22.5 mg; and high vitamin E intake was categorized as any intake above 22.5 mg.

Diet quality was assessed in all cohorts included in the secondary model by calculating the Mediterranean diet score [41]. Study-specific FFQs are detailed in the **Supplementary Materials**.

### DNA methylation

DNA methylation measurements were performed independently by each cohort. Fasting whole blood samples were collected from 11,866 participants. Samples for all cohorts except the Young Finns Study cohort were assayed using the Infinium HumanMethylation450 Beadchip (Illumina Inc., San Diego, CA), which covers >450,000 CpG sites. The Young Finns Study cohort samples were assayed using the MethylationEPIC 850K Beadchip (Illumina Inc., San Diego, CA), which covers >850,000 CpG sites. Each cohort normalized methylation beta values; details of the statistical methods for normalization are presented in the **Supplemental Materials**.

### Cohort-specific statistical models

Statistical models evaluated global associations of DNA methylation with vitamin intake by estimating the locus-by-locus association of self-reported vitamin intake with medium and high vitamin intake compared with low intake. All eight cohorts independently tested the association of dietary intake of vitamin C with DNA methylation using linear mixed models. Seven cohorts (the FHS Offspring study, the FHS Third-Generation study, the KORA study, the Young Finns study, the Raine Study, the Rotterdam Study, and the ARIC study) independently tested the association of dietary intake of vitamin E with DNA methylation using linear mixed models.

All statistical models were adjusted for age, sex, BMI, daily caloric intake, blood cell type proportion, smoking status (current/former/never), alcohol consumption, and assay-specific technical covariates. Blood cell type proportions (CD4+ T-cells, CD8+ T-cells, NK cells, monocytes, and eosinophils) were estimated for all participants based on DNA methylation values using the Houseman imputation method [42]. Cohort-specific technical covariates are detailed in the **Supplemental Materials**. To evaluate whether any significant associations were independent of overall healthy behaviour, a second model further adjusted for physical activity and diet quality. Data from all cohorts were included in both models with the exception of the KORA study cohort, which was not included in the second model due to unavailability of diet quality data at that time.

### Epigenome-wide meta-analysis

Inverse variance-weighted fixed-effects meta-analysis was used to combine cohort-specific results. Only CpG sites available in ≥3 cohorts were included in the meta-analysis: for the association between vitamin C intake and DNA methylation, 485,505 CpG sites were included in the meta-analysis, and for the association between vitamin E intake and DNA methylation, 485,433 CpG sites were included in the meta-analysis. The false discovery rate (FDR) was calculated for all models [43], and statistical significance was assessed at a FDR < 0.05.

### Discovery and replication

Due to differences in FFQs used to capture dietary and supplemental vitamin intake as well as potential differences in dietary patterns, we divided the total sample across cohorts into separate discovery and replication cohorts to investigate heterogeneity in the meta-analysis results and to assess the robustness of the topmost significant signals in our overall meta-analysis. We evaluated whether associations in the primary model were reproducible in the replication set. The discovery meta-analysis consisted of the 3,833 FHS Offspring and Third-Generation participants since both cohorts used the 126-item Harvard Willet FFQ and the same methods. The replication meta-analysis included 6,655 participants and consisted of the TwinsUK, Young Finns, the Rotterdam Study and ARIC study cohorts. Statistical significance at a FDR ≤ 0.05 was assessed in the replication cohort.

### Implications of DNA methylation: gene set enrichment analysis (GSEA) and expression quantitative trait methylation (eQTM) analysis

Genes to which CpG sites associated with vitamin intake were annotated were subsequently used in gene set enrichment analysis (GSEA) to identify putative biological process pathways directly affected by DNA methylation. CpG sites that were associated with vitamin intake at FDR ≤ 0.01 were included in gene ontology analysis using the ‘goana’ function in the R package *limma* [44]. Similarly, we conducted a second gene ontology analysis of all genes that were significantly associated with both vitamin C and E intake at FDR ≤ 0.05 to identify potentially shared pathways. Significant pathways in GSEA were identified at FDR ≤ 0.05.

CpG sites that were significantly associated with vitamin C or E intake at a FDR ≤ 0.01 were used in expression quantitative trait methylation (eQTM) analysis within the FHS cohort to identify downstream effects of DNA methylation on gene expression. eQTM analysis in the FHS has used DNA methylation data and RNA sequencing data to quantify the association between DNA methylation and gene expression independent of age, sex, white blood cell count, blood cell fraction, platelet count, genetic principal components (PCs), and DNA methylation PCs [45]. We identified significant *cis* associations between CpG sites and gene transcripts (i.e., associations where the CpG site was within 1Mb of the transcription start site) at a p-value of 1.0 × 10^−7^ [46]. For all significant *cis-*eQTM CpG-transcript pairs associated with vitamin C or vitamin E intake, we conducted a second GSEA of the gene transcripts to identify pathways that may be affected by vitamin intake-associated DNA methylation.

### Exploratory analyses: FHS discovery cohorts

Due to differences in methodology across cohorts, we conducted a series of exploratory and hypothesis-generating analyses in the discovery FHS Offspring and FHS Third-Generation cohorts. All statistical models described in this section were adjusted for all covariates in the primary model: age, sex, BMI, daily caloric intake, blood cell type proportion, smoking status, alcohol consumption, and assay-specific covariates.

#### Residualized analysis accounting for energy intake

We investigated the association between vitamin and total energy intake in a residual model to evaluate whether our main statistical models were fully accounting for total energy intake through statistical adjustment. Using this approach, we ran an initial regression model to calculate the residuals for the association between log-transformed continuous vitamin intake and total energy, and these residuals were used in a subsequent statistical model to quantify the association between vitamin intake and DNA methylation.

### Stratified analyses

To explore potential differences in DNA methylation by demographic, dietary, or behavioural factors, we performed several stratified analyses in the discovery cohort that combined both the FHS Offspring and FHS Third-Generation cohorts. We stratified the associations between DNA methylation and dietary intake of vitamins C and E by 1) male versus female sex; 2) age <65 years versus age ≥65 years; and 3) smoking status (current, former, and never smokers). Specifically, we ran regression models quantifying the adjusted association between DNA methylation and vitamin intake within each stratum of interest and identified CpG sites that were significantly associated with vitamin intake for each stratum at a FDR ≤ 0.05.

## Results

### Participant characteristics

Characteristics of participants from the study cohort are presented in Table 1.

**Table 1.** Participant characteristics by study cohort.

	FHS Offspring (*n* = 2,419)	FHS Third Generation (*n* = 1,441)	TwinsUK (*n* = 493)	KORA (*n* = 1,351)	Young Finns Study (*n* = 892)*	Raine Study (*n* = 512)	Rotterdam Study (*n* = 1,120)	ARIC Study, African ancestry (*n* = 2,570)	ARIC Study, European ancestry (*n* = 1,068)
Age	66 ± 9	45 ± 8	59 ± 9	58.3 ± 11.3	38 ± 5	17.0 ± 0.2	64 ± 8	53 ± 6	56 ± 5
Sex (n, % female)	1320 (55%)	758 (53%)	493 (100%)	717 (53.1%)	497 (56%)	277 (54%)	626 (56%)	1715 (64%)	626 (59%)
BMI (kg/m2)	28.2 ± 5.3	27.7 ± 5.7	26.6 ± 5.0	27.6 ± 5.0	25.7 ± 4.5	22.9 ± 4.2	27.5 ± 4.4	29.8 ± 6.0	25.8 ± 4.3
Caloric intake (kcal/day)	1880 ± 636	2059 ± 819	1871 ± 531	1851.9 ± 405.0	2392 ± 756	2233 ± 784	2202 ± 689	1571 ± 603	1538 ± 530
Vitamin C intake (n)									
Low (0-75 mg women, 0- 90 mg men)	380 (16%)	391 (27%)	20 (4%)	348 (26%)	130 (15%)	101 (20%)	293 (26%)	839 (33%)	343 (32%)
Medium (75- 300 mg women, 90- 300 mg men)	1445 (60%)	847 (59%)	445 (90%)	1002 (74%)	681 (76%)	366 (71%)	757 (68%)	1707 (66%)	706 (66%)
High (>300 mg)	594 (25%)	203 (14%)	28 (6%)	1 (0%)	28 (3%)	45 (9%)	70 (6%)	136 (5%)	19 (2%)
Vitamin E intake (n)									
Low (0-15 mg)	958 (40%)	907 (63%)	N/A^†^	1318 (98%)	747 (84%)	479 (59%)	662	2546 (99%)	1041 (97%)
Medium (15- 22.5 mg)	1050 (43%)	464 (32%)	N/A	33 (2%)	126 (14%)	32 (33%)	365	0	27 (3%)
High (>22.5 mg)	411 (17%)	70 (5%)	N/A	0 (0%)	19 (2%)^‡^	1 (8%)	93	24 (1%)	0

Note: *53 of 892 Young Finns Study participants did not have information on vitamin C intake and are not included in this breakdown.

*TwinsUK was not included in the vitamin E analyses and is not included in this breakdown.

^‡^Numbers presented in Table 1 are the mean ± SD for continuous variables and the n, % for categorical variables. Due to rounding, percentages may not add up to 100%.

### Vitamin C and DNA methylation

**Figure 2.**
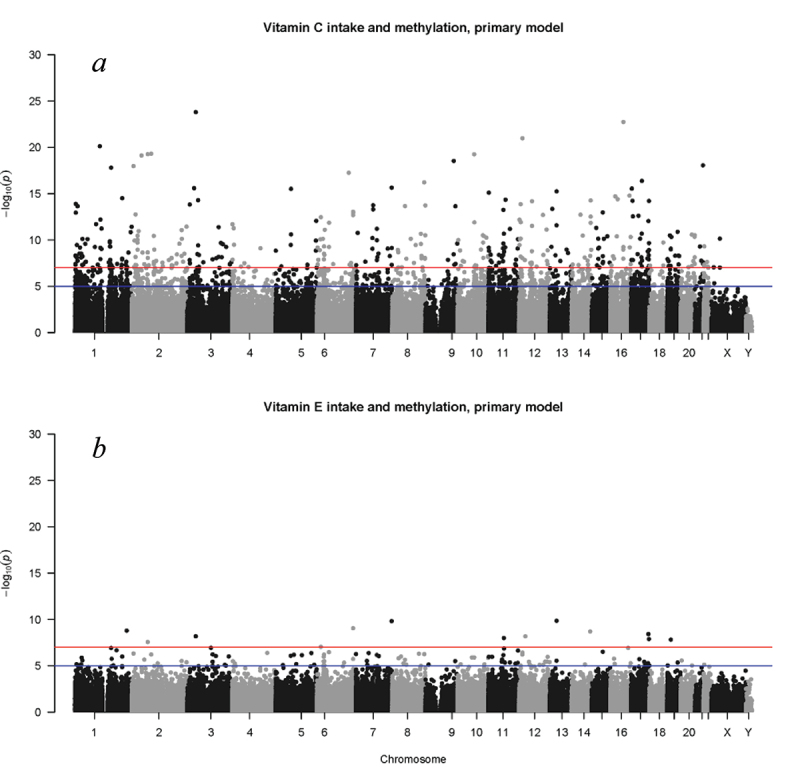
Manhattan plot of results of the meta-analysis including all study cohorts showing significant associations of Infinium HumanMethylation 450K BeadChip CpG sites with (a) vitamin C intake and (b) vitamin E intake after adjustment for age, sex, BMI, caloric intake, blood cell type proportion, smoking status, alcohol consumption, and technical covariates in the primary model. Blue and red horizontal lines indicate -log(p) values of 5 and 7, respectively (i.e., *p* = 1.0 × 10^−5^ and 1.0 × 10^−7^, representing a less stringent significance threshold for hypothesis generating and a more stringent approximately Bonferroni-corrected threshold, respectively).

In the meta-analysis combining EWAS results of all 8 cohorts, DNA methylation at 4,656 CpG sites was significantly associated with vitamin C intake after adjusting for age, sex, BMI, caloric intake, blood cell type proportions, smoking status, alcohol consumption, and technical covariates in the primary model (Figure 2a, Supplemental Table S2). These CpG sites were annotated to 2,586 unique genes. Vitamin C intake was associated with hypomethylation at 3,662 (79%) of these CpG sites, and with hypermethylation at the remaining 994 (21%) CpG sites. After further adjustment for diet quality and physical activity, vitamin C intake was associated with hypomethylation at 1,405 CpG sites that were annotated to 986 unique genes, and with hypermethylation at 389 CpG sites annotated to 267 unique genes (**Supplemental Table S3)**.

In discovery analyses, methylation at 3,643 CpG sites was significantly associated with vitamin C intake in the FHS cohorts after adjustment for all covariates in the primary model, of which 1,521 CpG sites overlapped with the significant results of the overall meta-analysis. Hypermethylation at 17 (0.5%) sites and hypomethylation at 256 (7%) sites was significantly associated with vitamin C intake in the meta-analysis of the replication cohorts at FDR ≤ 0.05 **(Supplemental Table S4).**

### Vitamin E and DNA methylation

DNA methylation at 160 CpG sites was significantly associated with vitamin E intake after adjusting for age, sex, BMI, caloric intake, blood cell type proportions, smoking status, alcohol consumption, and technical covariates in the primary model (Figure 2b, Supplemental Table S5). These CpG sites were annotated to 126 unique genes. Vitamin E intake was associated with hypomethylation at 115 (72%) of these CpG sites, and with hypermethylation at the remaining 45 (28%) CpG sites. After further adjustment for diet quality and physical activity, vitamin E intake was associated with hypermethylation at 17 CpG sites and with hypomethylation at 39 CpG sites (**Supplemental Table S6**).

In discovery analyses, methylation at 188 CpG sites was significantly associated with vitamin E intake in the FHS cohorts after adjustment for all covariates in the primary model, of which 81 CpG sites overlapped with the significant results of the overall meta-analysis. Hypomethylation of two of the CpG sites (1.1%) and hypermethylation of one of the CpG sites (0.5%) significantly associated with vitamin E intake in discovery analysis was replicated at FDR ≤ 0.05 **(Supplemental Table S7).**

In the primary model, DNA hypomethylation at 77 of the 160 CpG sites significantly associated with vitamin E intake was also significantly associated with vitamin C intake; similarly, DNA hypermethylation at eight CpG sites significantly associated with vitamin E intake was also significantly associated with vitamin C intake. After adjustment for diet quality and physical activity, 20 CpG sites were significantly associated with both vitamin C and E intake. Of these, 19 CpG sites were hypomethylated in association with both vitamin C and E and annotated to 18 unique genes (Table 2).

**Table 2.** EWAS meta-analysis results for CpG sites at which DNA hypomethylation was associated with both vitamin C and vitamin E intake at FDR ≤ 0.05 after adjusting for age, sex, BMI, caloric intake, blood cell type proportion, smoking status, alcohol consumption, technical covariates, physical activity, and diet.

		Association with vitamin C intake			Association with vitamin E intake		
CpG site	Gene Symbol	β ± SE, medium intake	β ± SE, high intake	FDR	β ± SE, medium intake	β ± SE, high intake	FDR
cg01894508	ASPRV1	−0.0041 ± 0.0009	−0.0069 ± 0.0013	2.7 × 10^−7^	−0.0028 ± 0.0011	−0.0044 ± 0.001	4.6 × 10^−2^
cg03084350	PLCD1	−0.0047 ± 0.0007	−0.006 ± 0.001	1.7 × 10^−12^	−0.0028 ± 0.0008	−0.0033 ± 0.0008	3.0 × 10^−2^
cg08051604	MEG3	−0.002 ± 0.0008	−0.0056 ± 0.0013	3.1 × 10^−3^	−0.0035 ± 0.0011	−0.0049 ± 0.0011	8.5 × 10^−3^
cg09121339		−0.0055 ± 0.0009	−0.0072 ± 0.0013	6.3 × 10^−11^	−0.0028 ± 0.0011	−0.0042 ± 0.001	3.2 × 10^−2^
cg09387914	ELOVL5	−0.0028 ± 0.0009	−0.005 ± 0.0014	7.7 × 10^−3^	−0.0038 ± 0.0012	−0.0049 ± 0.0011	1.4 × 10^−2^
cg10401362	DNAJB6	−0.0045 ± 0.001	−0.006 ± 0.0015	2.1 × 10^−5^	−0.0061 ± 0.0012	−0.0043 ± 0.0012	2.1 × 10^−3^
cg11027221	UNC93B1	−0.003 ± 0.0008	−0.0046 ± 0.0011	1.2 × 10^−4^	−0.003 ± 0.0009	−0.0036 ± 0.0008	8.5 × 10^−3^
cg11348106	SEC14L1	−0.0032 ± 0.0009	−0.0034 ± 0.0014	4.6 × 10^−2^	−0.0048 ± 0.0012	−0.0035 ± 0.0011	2.9 × 10^−2^
cg11879354	SYTL3	−0.003 ± 0.0006	−0.0044 ± 0.0009	5.2 × 10^−6^	−0.0019 ± 0.0008	−0.004 ± 0.0007	2.1 × 10^−3^
cg12061917	GABBR1	−0.0036 ± 0.0009	−0.0043 ± 0.0012	1.1 × 10^−3^	−0.0025 ± 0.001	−0.0041 ± 0.001	3.9 × 10^−2^
cg14150666	CXCR2	−0.0036 ± 0.0011	−0.0055 ± 0.0017	1.0 × 10^−2^	−0.0043 ± 0.0015	−0.0057 ± 0.0014	3.8 × 10^−2^
cg14781394		−0.0027 ± 0.0008	−0.0058 ± 0.0012	1.6 × 10^−4^	−0.0035 ± 0.001	−0.0042 ± 0.001	8.5 × 10^−3^
cg15866367	MLNR	−0.003 ± 0.0008	−0.0044 ± 0.0013	3.0 × 10^−3^	−0.0049 ± 0.0011	−0.0041 ± 0.0011	2.1 × 10^−3^
cg18818531	FOSL1	−0.0037 ± 0.0008	−0.0061 ± 0.0012	5.0 × 10^−7^	−0.0032 ± 0.001	−0.0035 ± 0.0009	2.5 × 10^−2^
cg20761853	TIMP2	−0.0029 ± 0.001	−0.006 ± 0.0015	3.7 × 10^−3^	−0.0035 ± 0.0013	−0.0055 ± 0.0012	2.5 × 10^−2^
cg22753611	CAP2	−0.0032 ± 0.0011	−0.0062 ± 0.0016	5.1 × 10^−3^	−0.0033 ± 0.0013	−0.0059 ± 0.0013	1.5 × 10^−2^
cg23633330	CD55	−0.004 ± 0.0007	−0.0056 ± 0.0011	3.6 × 10^−8^	−0.0026 ± 0.0009	−0.0036 ± 0.0009	3.9 × 10^−2^
cg25513379	DOCK2;FAM196B	−0.0026 ± 0.0007	−0.0026 ± 0.0011	4.1 × 10^−2^	−0.0045 ± 0.001	−0.0028 ± 0.0009	9.6 × 10^−3^
cg26250129	SLC38A10	−0.0041 ± 0.0009	−0.0062 ± 0.0013	7.9 × 10^−7^	−0.0032 ± 0.0011	−0.0046 ± 0.001	8.5 × 10^−3^

### Downstream pathways affected by DNA methylation: eQTM and GSEA

At FDR ≤ 0.01, there were 1,907 CpG sites significantly associated with vitamin C in the primary model. These CpG sites were annotated to 1,163 unique genes that were significantly enriched in 673 pathways associated with system development and cell signalling and communication **(Supplemental Table S8)**. These 1,907 CpG sites were also associated with expression of 425 unique gene transcripts in eQTM analysis (**Supplemental Table S9**). The gene transcripts identified by eQTM analysis were enriched in 44 biological pathways that were largely related to the immune response (**Supplemental Table S10**).

At FDR ≤ 0.01, there were 37 CpG sites that were significantly associated with vitamin E intake in the primary model. These CpG sites were annotated to 29 genes; these genes were not enriched in any cellular or biological pathways. These 37 CpG sites were also associated with the expression of 9 genes (**Supplemental Table S11)**. These genes were not enriched in any biological pathways at an enrichment FDR ≤ 0.05.

### Downstream pathways affected by DNA methylation: CpG sites associated with both vitamin C and vitamin E intake

The 19 CpG sites at which DNA methylation was associated with intake of both vitamins C and E were not enriched in any pathways in GSEA.

### Exploratory analyses in the discovery FHS cohorts

#### Residualized analyses

Inclusion of the residualized association between vitamin intake and total energy intake revealed 261 significant associations between continuous vitamin C intake and DNA methylation **(Supplemental Table S12)**, where hypomethylation was associated with vitamin C intake at 227 CpG sites and hypermethylation was associated with vitamin C intake at 34 CpG sites. Similarly, residualized analysis revealed 26 significant associations between continuous vitamin E intake and DNA methylation (**Supplemental Table S13**), where hypomethylation was associated with vitamin E intake at 23 CpG sites and hypermethylation was associated with vitamin E intake at 3 CpG sites.

#### Sex-stratified results

In men, DNA hypomethylation at 78 CpG sites was significantly associated with vitamin C intake and DNA hypermethylation at 39 CpG sites was significantly associated with vitamin C intake (**Supplemental Table S14)**. In women, DNA hypomethylation at 40 CpG sites and hypermethylation at 11 CpG sites were associated with vitamin C intake (**Supplemental Table S15)**. Only two CpG sites were associated with vitamin C intake in both men and women: cg03084350 (*PLCD1)* and cg09018739 (*CPNE2*).

In men, there were no significant associations between DNA methylation and vitamin E intake; however, in women, DNA hypomethylation at three CpG sites was significantly associated with vitamin E intake (**Supplemental Table S16).**

#### Age-stratified results

In participants aged 65 years and older, DNA hypomethylation at 32 CpG sites and hypermethylation at 24 CpG sites was associated with vitamin C intake **(Supplemental Table S17)**. In participants under the age of 65 years, DNA hypomethylation at 73 CpG sites and hypermethylation at 11 CpG sites was associated with vitamin C intake **(Supplemental Table S18)**. Four CpG sites were associated with vitamin C intake in both age strata.

In participants aged 65 years and older, DNA hypomethylation at two CpG sites and hyper-methylation at one CpG site were associated with vitamin E intake (**Supplemental Table S19)**. In participants under the age of 65 years, DNA hypomethylation at six CpG sites and hypermethylation at one CpG site were associated with vitamin E intake (**Supplemental Table S20)**. No CpG sites were associated with vitamin E intake in both age strata.

#### Smoking status-stratified results

In never smokers, DNA hypomethylation at seven CpG sites and hypermethylation at 11 CpG sites were associated with vitamin C intake (**Supplemental Table S21)**. In former smokers, DNA hypomethylation at 64 CpG sites and hypermethylation at 16 CpG sites were associated with vitamin C intake (**Supplemental Table S22)**. Finally, in current smokers, DNA hypomethylation at four CpG sites and hypermethylation at five CpG sites were associated with vitamin C intake **(Supplemental Table S23).**

DNA methylation was not significantly associated with vitamin E intake at any CpG sites in never or in current smokers. DNA hypomethylation was significantly associated with vitamin E intake at one CpG site only – cg13574809 – in former smokers (**Supplemental Table S24)**.

## Discussion

In this study, we combined data across multiple large population-based cohorts to examine the association of vitamin C and E intake with DNA methylation signatures. We provide evidence that greater intake of vitamins C and E is associated with DNA methylation signatures, and identified several thousand CpG sites associated with vitamin C intake, and approximately 100 CpG sites associated with vitamin E intake. The majority of these sites were hypomethylated in relation to increased vitamin intake, even in stratified analysis; furthermore, DNA methylation at the CpG sites identified in EWAS was inversely associated with gene expression in subsequent eQTM analysis.

Oxidative stress affects cellular and biological processes, and it can lead to inflammation and development of clinical disease, including atherosclerosis and metabolic disease [47–50]. Antioxidants from a healthy diet, supplementation, or pharmacological interventions may improve immune response and prevent oxidative stress-related cardiometabolic disease. In humans, vitamin C must be obtained exogenously either through diet (e.g., citrus fruit, cruciferous vegetables, tomatoes), supplementation, or pharmacological administration. It acts as a cofactor of TET proteins, which catalyse the hydroxylation of methyl groups at the cytosine C5 position for subsequent removal in DNA demethylation. Conversely, vitamin E is a lipid-soluble antioxidant found in cell membranes that interrupts radical chain reactions and may subsequently prevent inflammation [21,51,52]. It interacts with vitamin C to subsequently become reduced back into active vitamin E and then continue acting as an antioxidant in the cell membrane. Similar to vitamin C, it must be obtained through exogenous means (e.g., consumption of seeds, nuts, and oils). Prior studies have suggested a role of vitamin intake-associated DNA methylation in the expression of particular genes, such as the *ABCA1* gene [53] or genes associated with oxidative stress [54] We identified 85 CpG sites at which DNA methylation was significantly associated with both vitamin C and vitamin E intake, of which 20 CpG sites had an association that was independent of other lifestyle or behavioural risk factors. Although enrichment analysis did not reveal a pathway in which the genes annotated to these CpG sites DNA methylation at these sites may play a role, this overlap is suggestive of the known antioxidative interaction between vitamins C and E. Future studies may evaluate the cellular and biological effects of suppressing the genes associated with intake of both vitamins, as our results showed predominant associations of increased dietary vitamin intake with DNA hypomethylation.

While the observed differences in our stratified analyses were limited by reduced power in the discovery cohort, we generally saw that there were differences in which CpG sites were associated with vitamin intake by age, sex, and smoking status, particularly in association with vitamin C intake. In summary, men, individuals less than 65 years of age, and former smokers had the greatest number of significant associations between DNA methylation and vitamin C intake as compared with women, individuals over 65 years of age, never smokers, and current smokers. Like the global meta-analysis results, vitamin C intake was mostly associated with DNA hypomethylation among all stratified groups: hypomethylation at 67%, 87%, and 80% of CpG sites significantly associated with vitamin C intake in men, individuals younger than 65 years, and former smokers, respectively. These preliminary results may reflect a more active role for vitamin C in the setting of higher oxidative stress (i.e., in men and in former smokers) or without accounting for other behaviours potentially found in adults younger than 65 years. Further studies may focus on expanding these stratified analyses to larger cohorts.

Our results further suggest a role for vitamin C-associated DNA methylation patterns in activation of the immune response. We identified numerous biological pathways potentially directly affected by vitamin C-associated methylation: response to stimulus, regulation of biological and cellular processes, and cell signalling and communication. Further analysis of eQTM CpG-gene transcript pairs showed that the DNA methylation patterns associated with vitamin C intake are associated with the downstream expression of genes that are in the immune response pathway. Vitamin E has also previously been shown to be associated with the inflammatory response [55–57]; however, while prior studies have shown an association between vitamin E concentration and expression of the *DNMT1* gene [58,59], we were not able to identify any gene ontology pathways associated with vitamin E-associated DNA methylation.

While this study benefits from the availability of data from large population-based cohorts to evaluate the association of vitamin intake with both DNA methylation and gene expression, several limitations must be considered when interpreting and applying our results. Firstly, as these data were collected as part of large observational studies, causal inference is not possible; future mediation analyses evaluating the relationship between dietary vitamin intake, DNA methylation, and gene expression will reveal potential causal mechanisms. Our meta-analysis included only individuals with European and African ancestry, and generalizability of these findings to other populations may be limited. While the majority of participants included in our meta-analysis were adults, the 512 participants enrolled in the Raine Study (4% of the total participants included in the main meta-analysis) were 17 years of age at the time of data collection. Individuals below the age of 18 have different vitamin C and E recommended dietary allowance thresholds as compared with adults over the age of 18. Additionally, when we split the overall sample for our main analysis into a discovery cohort and a replication cohort, we observed poor replication for both the vitamin C and the vitamin E analyses, potentially due to major differences in how these FFQs captured and reported vitamin intake and consequent high heterogeneity across cohorts for some CpG sites. Dietary intake of vitamins C and E was based solely on self-reported dietary intake rather than on a biomarker measure, and we did not include analysis of any other antioxidants in our analysis, preventing analysis of their potential effects on DNA methylation. We also used categorical vitamin intake based on clinically relevant thresholds in our main analyses, although we completed sensitivity analyses that residualized total energy intake by continuous vitamin intake in our discovery cohort. However, due to non-normal distributions of self-reported vitamin intake, our approach allows for easier interpretation of the association between levels of vitamin intake and DNA methylation, and it is consistent across all included cohorts. Our definition of vitamin C intake included the use of supplements, and supplemental intake of multivitamins may introduce confounding due to the presence of folic acid and other nutrients that are associated with DNA methylation. Prior analyses have shown few significant associations between DNA methylation and folate intake [60,61], and so we expect these nutrients not to influence our observed results greatly. While we adjusted for diet quality using the Mediterranean diet score to account for overall healthy diets, simultaneous supplemental intake of multiple vitamins and nutrients may not be adequately captured. Furthermore, while our secondary model did adjust for lifestyle and behavioural risk factors by accounting for diet quality and physical activity level, we did not have data on and thus could not account for environmental exposures that are known to be associated with DNA methylation patterns.

In conclusion, dietary and supplementary intake of vitamin C and vitamin E is associated with DNA methylation, which likely plays a critical role in the regulation of cell signalling mechanisms and subsequently, in the association of vitamin intake with the immune response. Our results highlight the importance of dietary and supplementary intake of vitamins C and E through consumption of fruit, vegetables, nuts, seeds, and oils in overall health and the reduction of burden from inflammatory processes.

## Supplementary Material

Supplemental Material

## Data Availability

FHS: Data from the FHS Offspring and Third-Generation cohorts are available at the Database of Genotypes and Phenotypes (dbGaP) at https://www.ncbi.nlm.nih.gov/gap/, reference number phs000007.v29.p10. TwinsUK: Many of the data analysed in the current study is available through GEO GSE62992 and GSE121633. Additional individual-level data are not permitted to be shared or deposited due to the original consent given at the time of data collection. However, access to these data can be applied for through the TwinsUK data access committee. For information on access and how to apply http://twinsuk.ac.uk/resources-for-researchers/access-our-data/. KORA: The informed consents given by KORA study participants do not cover data posting in public databases. However, data are available upon request from KORA Project Application Self-Service Tool (https://helmholtz-muenchen.managed-otrs.com/external) Data requests can be submitted online and are subject to approval by the KORA Board. Young Finns Study: The dataset supporting the conclusions of this article were obtained from the Cardiovascular Risk In Young Finns study which comprises health-related participant data. The use of data is restricted under the regulations on professional secrecy (Act on the Openness of Government Activities, 612/1999) and on sensitive personal data (Personal Data Act, 523/1999, implementing the EU data protection directive 95/46/EC). Due to these restrictions, the data can not be stored in public repositories or otherwise made publicly available. Data access may be permitted on a case by case basis upon request only. Data sharing outside the group is done in collaboration with YFS group and requires a data-sharing agreement. Investigators can submit an expression of interest to the chairman of the publication committee (Prof Mika Kähönen, Tampere University, Finland) or Professor Terho Lehtimäki (Tampere University, Finland) concerning the epigenetic and genetic data. Raine Study: The Raine Study holds a rich and detailed collection of data gathered over 30 years for the purpose of health and well-being research. The informed consent provided by each participant does not permit individual-level data to be made available in the public domain (i.e., a public data repository). However, de-identified analytic data sets are available to all researchers for original research or auditing of published findings. All data access is managed through established Raine Study procedures which require data handlers to agree to a code of conduct that includes safeguards to protect the identity of participants. Details of the data access processes, and code of conduct are available on the Raine Study website https://rainestudy.org.au/. The governance process for accessing the Raine Study data can be accessed here: https://rainestudy.org.au/information-for-researchers/information-for-new-researchers/. Rotterdam Study: The Rotterdam Study data can be made available to interested researchers upon reasonable request. Requests can be directed to data manager Frank J.A. van Rooij (ln.cmsumsare@jioornav.f). We are unable to place data in a public repository due to legal and ethical restraints. Sharing of individual participant data was not included in the informed consent of the study, and there is potential risk of revealing participants’ identities as it is not possible to completely anonymize the data. This is of particular concern given the sensitive personal nature of much of the data collected as part of the Rotterdam Study. ARIC Study: The DNA methylation dataset from ARIC is available upon request at https://sites.cscc.unc.edu/aric/distribution-agreements.
