# Supplementary material for: Dietary and supplemental intake of vitamins C and E is associated with altered DNA methylation in an epigenome-wide association study meta-analysis
Source: Epigenetics. 2023 May 26;18(1):2211361. doi: 10.1080/15592294.2023.2211361 (PMC10228397; doi:10.1080/15592294.2023.2211361)
Supplement: Supplemental Material [file KEPI_A_2211361_SM4908.zip › Supplementary files/vitce_suppl_methods_05.docx]

# Supplementary Materials: Cohort-specific methods

## Framingham Heart Study (FHS): Offspring and Third Generation cohorts

### Cohort description

The FHS is a longitudinal population-based cohort study. The present analyses included FHS Offspring cohort participants who attended the eighth examination (2005-2008) and FHS Third Generation cohort participants who attended the second examination (2008-2011). All study protocols, including for participant examinations and for the collection and storage of genetic materials, were approved by the Boston University Medical Center Institutional Review Board.

### Clinical data

Participants underwent a clinical examination that included collection of a blood sample, anthropometric measures, and medical and behavioral information. Alcohol intake was captured by asking participants about their weekly consumption of beer, wine, and 80-proof spirits in the past year. A physical activity index was derived from self-reported time spent doing light, moderate, and heavy activity, being sedentary, and sleeping.^1^

### Diet

Participants completed the validated 126-item Harvard Willet Food Frequency Questionnaire (FFQ) prior to their in-person examination.^2,3^ These data were used to estimate daily caloric intake, vitamin C intake (in milligrams per day), and vitamin E intake (in international units [IU] per day). Supplemental multivitamin intake was captured as a dichotomous yes/no variable and used to calculate overall vitamin intake.

### DNA methylation

Samples from study participants with leukemia and from those who underwent chemotherapy were excluded from these analyses. Buffy coats were isolated from fasting peripheral whole blood samples. DNA was collected using the Gentra Puregene DNA extraction kit (Qiagen, Hilden, Germany) and bisulfite converted using the EZ DNA methylation kit (Zymo Research, Irvine, CA). DNA methylation was assayed using the Infinium HumanMethylation 450 Beadchip (Illumina Inc., San Diego, CA).

Raw methylation β values were normalized using the dasen function in the R package *wateRmelon*^4^ and underwent background correction using the preprocessIllumina function in the R package *minfi.*^5,6^ The normalized β methylation value for analysis was calculated as the proportion of DNA methylation per CpG site, and was evaluated for all included samples at 485,512 CpG sites.

Subsequent exclusions were made to samples with poor SNP matching of control positions, with missing DNA methylation data at a rate > 1%, where the reported and methylation-predicted sex did not match, where the methylation-predicted tissue was not blood, and where the correlation with the reference population < 80% were excluded from analysis. Technical covariates included in the EWAS linear regression models included row, column, chip, and principal components (PCs).

## UK Adult Twin Registry (TwinsUK)

### Cohort description

The TwinsUK registry cohort recruited monozygotic and dizygotic same-sex twins in the United Kingdom beginning in 1992.^7^ The present study included 493 female, Caucasian twins from the original registry cohort.

### Clinical data

These analyses included data from TwinsUK participants who completed a comprehensive clinical exam during which they provided biological specimens and completed questionnaires to provide self-reported data on demographic, lifestyle, and behavioral outcomes. Physical activity data were not evaluated in this study. All study participants provided their written informed consent.

### Diet

TwinsUK registry participants completed the European Prospective Investigation into Cancer and Nutrition (EPIC)-Norfolk FFQ,^8,9^ a validated semi-quantitative FFQ where participants report consumption for 131 food and drink items. Participant responses were analyzed using the FFQ EPIC Tool for Analysis (FETA) to obtain nutrient levels, including milligrams of vitamin C and vitamin E intake. Daily alcohol intake in TwinsUK was estimated directly from the food frequency questionnaires using this same methodology.

### DNA methylation

DNA samples were extracted from peripheral whole blood samples using the DNeasy kit (Qiagen) and bisulfite converted using the EZ DNA methylation kit (Zymo Research). DNA methylation was assayed using the Illumina Infinium HumanMethylation450 BeadChip (Illumina Inc., San Diego, CA). Methylation beta values were obtained using the R package *minfi*^5^ for background correction, and the ENmix pipeline^10,11^ was applied to correct for technical effects, including quantile normalization of signals and probe design bias correction with the RCP method.^12^ Signals with a detection p-value >0.000001 and number of beads <3 were excluded from the analysis, as well as samples and probes with >5% missingness. DNA methylation data were used to predict sex, blood cell types, age, and to compute correlations with the reference population.^13^ Ultimately, 482,668 CpG sites were included in these analyses. The EWAS regression models were adjusted for the following technical covariates as random effects: plate, plate position, family structure, and zygosity structure. All twin participants were females and sex was not included in the TwinsUK regression models.

## Kooperative Gesundheitsforschung in der Region Augsburg (KORA)

### Cohort description

The KORA study is a population-based cohort study from Augsburg, Southern Germany. A total of 4,261 participants completed the baseline S4 survey in 1999-20001. Out of these 2,279 individuals participated in the second follow-up study FF4 in 2013-2014. The present analyses included data from 1,351 FF4 participants. Study participants provided written informed consent, and the study was approved by the Ethics Committee of the Bavarian Medical Association (Bayerische Landesärztekammer).

### Clinical data

All FF4 participants completed an in-person interview and a physical examination by trained staff during which anthropometric, laboratory, and clinical data were collected.

### Diet

Participants completed at least two 24-hours recalls and one FFQ.^14,15^ Consumption probabilities were estimated using logistic mixed models, adjusting for covariates and the FFQ data on consumption frequency. Dietary intake amount of a consumed food item was predicted for each participant based on the results of the second Bavarian Food Consumption Survey (BVS II). By combining consumption probability and estimated consumption amount, the usual food intake for each participant was estimated. The German food composition table BLS III (version 3.02) was used to calculate energy and nutrient intake data, including intake of vitamin C and E.^7^ Intake of dietary supplements during the last 7 d was assessed together with medication use through computer-based software in a personal interview (LIT).^16^ The term “supplemental” vitamin intake captures both intake from supplements and from medications as the same ingredients (e.g. a-tocopherol) can be marketed either as supplements or as medications in Germany. Supplemental vitamin intake was captured by three variables: whether a supplement was taken in the prior seven days; regularity of supplement intake (never vs. when needed vs. regularly); and daily amount of supplemental vitamin intake in milligrams.

### DNA methylation

Genomic DNA (750 ng) from 1928 individuals was bisulfite converted using the EZ-96 DNA Methylation Kit (Zymo Research, Orange, CA, USA) in two separate batches (N=488, N=1440). Subsequent methylation analysis was performed on an Illumina (San Diego, CA, USA) iScan platform using the Infinium MethylationEPIC BeadChip according to standard protocols provided by Illumina. GenomeStudio software version 2011.1 with Methylation Module version 1.9.0 was used for initial quality control of assay performance and for generation of methylation data export files.

Further quality control and preprocessing of the data were performed in R v3.5.1 (R Core Team (2017). R: A language and environment for statistical computing. R Foundation for Statistical Computing, Vienna, Austria. URL <https://www.R-project.org/>), with the package *minfi* v1.28.3^5^ and following primarily the CPACOR pipeline.^17^ Raw intensities were read into R (command read.metharray) and background corrected (bgcorrect.illumina). Probes with detection p-values >0.01 were set to missing.

Before normalization, we removed problematic samples and probes. Forty samples were removed: 2 showed a mismatch between reported sex and that predicted by minfi; 33 had median intensity <50% of the experiment-wide mean, or <2000 arbitrary units; and 9 (overlap of 4 with previous) had >5% missing values on the autosomes. A total of 59631 probes were removed (some overlapping multiple categories): cross-reactive probes as given in published lists^18,19^ (N=44,493); probes with SNPs with minor allele frequency >5% at the CG position (N=11,370) or the single base extension (N=5597) as given by minfi; and 5786 with >5% missing values. A total of 806,228 probes remained for analysis. Quantile normalization (QN) was then performed separately on the signal intensities divided into the 6 probe types: type II red, type II green, type I green unmethylated, type I green methylated, type I red unmethylated, type I red methylated. To adjust for technical variation, the principal components of the (non-negative) control probes were determined and the top 20 were used as covariates in the models, as per the CPACOR pipeline.

As methylation levels in blood can be strongly influenced by leukocyte composition, the white blood cell type proportions were calculated using the method of Houseman et al.^20^ as implemented in minfi (command estimateCellCounts, default parameters on the raw data) and used as covariates in the models.

Data from the KORA study were not included in the vitamin C or E secondary models that adjusted for physical activity and diet quality.

## Young Finns study (YFS)

### Cohort description

The YFS enrolled 3,596 children between the ages of 3 and 18 years in 1980. The present analyses included data from 892 YFS participants who completed follow-up assessments in 2007.

### Clinical data

Participants completed a clinical examination during the 2007 follow-up visit. A physical activity index was calculated as metabolic equivalents (METs) based on frequency, intensity, and duration of self-reported physical activity. The YFS was approved by the Ethical Review Committee of Turku University Hospital and complied with the Declaration of Helsinki. All study participants provided their written informed consent.

### Diet

A 131-item FFQ developed by the Finnish National Institute for Health and Welfare was administered to capture habitual dietary intake over the prior year.^21^ Nutrient, caloric, and alcohol intake were calculated using the Finnish Food Composition database (<https://fineli.fi/fineli/en/index>).

### DNA methylation

DNA was isolated from whole blood using a Wizard® Genomic DNA Puridivation Kit (Promega Corporation, Madison, WI, USA). Genome-wide DNA methylation was assayed using the Illumina Infinium MethylationEPIC array and data were pre-processed using the *minfi* R package.^5,6^ Background correction and dye-bias normalization was performed using noob^22^ and stratified quantile normalization. Probes where the detection p-value was > 0.01 in 99% of samples, that were cross-reactive, and that were SNPs were excluded.

This yielded a total of 787,044 CpG sites for which there was available YFS data; of these, 407,414 CpG sites overlapped with the Illumina Infinium HumanMethylation450 BeadChip and were used in these analyses. The EWAS models adjusted for batch effects by adjusting for the first 30 control probe-based PCs.

## Raine study

### Cohort description

The Raine study is a multigenerational longitudinal cohort study that enrolled 2,900 pregnant women who were between 16 and 20 weeks gestation in Perth, Australia (1989-1990). The offspring of the original cohort (Generation 2, n=2,868) have been followed for 17 years. The present study included data from 512 Generation 2 participants who were aged 17 years.

### Clinical data

Raine Study participants completed follow-up visits at which anthropometric and clinical data were collected. Participants provided blood specimens at their 17-year follow-up visit. The study was approved by the Human Ethics Committee of the University of Western Australia. All participants and their parents provided written informed consent.

### Diet

Study participants completed a semi-quantitative FFQ that was developed by the Commonwealth Scientific and Industrial Research Organisation (CSIRO) in Adelade, Australia.^23^ The FFQ evaluated consumption of 212 foods and was validated in the Raine study cohort against a 3-day food record.^24^ Nutrient and caloric intake was estimated using Australian food composition data (<https://www.foodstandards.gov.au/science/monitoringnutrients/afcd/Pages/default.aspx>).

### DNA methylation

DNA methylation was assayed on whole blood samples using the Illumina Infinium HumanMethylation450 BeadChip array (Illumina Inc., San Diego, CA). Quality control was completed in R using the packages *shinyMethyl,*^25^ *MethylAid,*^26^ and *RnBeads.*^27^ BMIQ was applied to the DNA methylation data for normalization. Samples from participants with inconsistent results or sex misclassification were excluded.

Probes for SNPs, on allosomal chromosomes, or with a detection p-value > 0.05 in at least one sample were excluded. Technical covariates included in EWAS models included plate, slide, and well number. Each CpG was mapped to the nearest gene using the Illumina Infinium annotation coordinates, and a total of 474,481 probes were included in the EWAS.

## Rotterdam Study (RS)

### Cohort description

The RS is a population-based cohort study that recruited residents of the district Ommoord in Rotterdam, the Netherlands, who were aged 45 and older. The present study included 417 participants from cohort 2 visit 3 (RS-II-3), 531 participants from cohort 3 visit 1 (RS-III-1), and 172 participants from cohort 3 visit 2 (RS-III-2).

### Clinical data

Self-reported data on tobacco smoking was collected from questionnaires and used to define smoking status. Anthropometric measures and blood collections was performed at the research center following standard procedures. All participants provided written informed consent. Physical activity was assessed with the Longitudinal Aging Study Amsterdam (LASA) physical activity questionnaire (LAPAQ). Metabolic equivalents (METs) were assigned to activities captured by the questionnaire according to the 2011 Compendium of Physical Activities.^28^ MET values and self-reported frequency of physical activity were used to calculate the hours of physical activity per week for inclusion in the EWAS statistical models.

### Diet

Study participants completed a semi-quantitative 389-item Dutch FFQ.^29,30^ Nutrient and caloric intake were estimated using the 2011 Dutch Food Composition Database (<https://www.rivm.nl/en/dutch-food-composition-database>).

### DNA methylation

Whole blood samples were obtained and stored in EDTA tubes and DNA was extracted using a salting out method. Purified DNA was subsequently bisulfite treated using the Zymo EZ96 DNA methylation kit (Zymo Research, Irvine, CA). Samples with incomplete bisulfite treatment, with a detection rate < 99%, and with mismatched sex were excluded. Genome-wide DNA methylation was measured using the Illumina Infinium HumanMethylation450 BeadChip assay (Illumina Inc., San Diego, CA), and the DNA methylation β value was calculated as the methylation proportion for each CpG site. Probes with a detection p-value > 0.01 in at least 1% of samples were excluded from analysis.

Technical covariates for the EWAS included array number and array position, and these were modeled as random effects. 473,682 CpG sites were included in the final analysis.

## Atherosclerotic Risk in Communities (ARIC) Study

### Cohort description

The ARIC study is a prospective cohort study that recruited 15,792 men and women between the ages of 45 and 64 years from four study sites in the United States: Forsyth County, North Carolina; Jackson, Mississippi; Minneapolis, Minnesota; and Washington County, Maryland. Study participants completed an initial examination (Visit 1) between 1987 and 1989. The present study included data from 2,570 ARIC participants of self-reported African (AA) and 1,068 ARIC participants of European (EA) race/ethnicity.

### Clinical data

Each study visit included a comprehensive clinical exam where anthropometric, clinical, and demographic data were collected. Current cigarette smoking and current alcohol consumption were collected via questionnaire. Physical activity was measured by a modified self-administered Baecke questionnaire^31^ to assess three domains of habitual physical activity (sports, leisure time, and work) on a scale of 1 (low) to 5 (high) {PMID: 9243489}. This scale was this dichotomized so that the 20% of men and women with the lowest age-adjusted habitual physical activity score were coded as physically inactive, while everyone on else was coded as physically active for each self-reported race/ethnicity separately as previously described in Graff et al.^32^ All study protocols were approved by the institutional review board of each study site, and written informed consent was obtained.

### Diet

The ARIC study FFQ is a 66-item questionnaire derived from the 61-item Willett FFQ.^33,34^ Participants reported average frequency of consumption of various food types over the prior year. Nutrient and caloric intake were estimated at the Channing Laboratory at Harvard Medical School.

### DNA methylation

DNA was extracted from peripheral blood samples using the Gentra Puregene Blood Kit (Qiagen, Valencia, CA). Genomic DNA was bisulfite converted using the EZ-96 DNA Methylation Kit (Zymo Research, Irvine, CA). Efficiency of the bisulfite conversion was evaluated via PCR amplification of the converted DNA, and DNA methylation was subsequently assayed using the Illumina Infinium HumanMethylation450 BeadChip array. Raw methylation values were normalized using BMIQ. Samples with a detection p-value > 0.01 for at least 1% of CpG sites or with potential sex mismatch were excluded from analysis. CpG sites with an average detection p-value > 0.01 for at least 1% of samples were similarly excluded.

All EWAS models were run separately for the ARIC sample with AA and the ARIC sample with EA. 483,735 CpG sites were available for analysis in the AA sample, and 482,847 CpG sites were available for analysis in the EA sample.

## References

1. Kannel WB, Sorlie P. Some Health Benefits of Physical Activity: The Framingham Study. *Arch Intern Med*. 1979;139(8):857-861. doi:10.1001/archinte.1979.03630450011006

2. Rimm EB, Giovannucci EL, Stampfer MJ, Colditz GA, Litin LB, Willett WC. Reproducibility and validity of an expanded self-administered semiquantitative food frequency questionnaire among male health professionals. *Am J Epidemiol*. 1992;135(10):1114-1136. doi:10.1093/oxfordjournals.aje.a116211

3. Jacques PF, Sulsky SI, Sadowski JA, Phillips JC, Rush D, Willett WC. Comparison of micronutrient intake measured by a dietary questionnaire and biochemical indicators of micronutrient status. *Am J Clin Nutr*. 1993;57(2):182-189. doi:10.1093/ajcn/57.2.182

4. Pidsley R, Y Wong CC, Volta M, Lunnon K, Mill J, Schalkwyk LC. A data-driven approach to preprocessing Illumina 450K methylation array data. *BMC Genomics*. 2013;14(1):293. doi:10.1186/1471-2164-14-293

5. Aryee MJ, Jaffe AE, Corrada-Bravo H, et al. Minfi: a flexible and comprehensive Bioconductor package for the analysis of Infinium DNA methylation microarrays. *Bioinformatics*. 2014;30(10):1363-1369. doi:10.1093/bioinformatics/btu049

6. Fortin JP, Triche Jr TJ, Hansen KD. Preprocessing, normalization and integration of the Illumina HumanMethylationEPIC array with minfi. *Bioinformatics*. 2017;33(4):558-560. doi:10.1093/bioinformatics/btw691

7. Moayyeri A, Hammond CJ, Valdes AM, Spector TD. Cohort Profile: TwinsUK and healthy ageing twin study. *Int J Epidemiol*. 2013;42(1):76-85. doi:10.1093/ije/dyr207

8. Bingham SA, Welch AA, McTaggart A, et al. Nutritional methods in the European Prospective Investigation of Cancer in Norfolk. *Public Health Nutr*. 2001;4(3):847-858. doi:10.1079/phn2000102

9. McKeown NM, Day NE, Welch AA, et al. Use of biological markers to validate self-reported dietary intake in a random sample of the European Prospective Investigation into Cancer United Kingdom Norfolk cohort. *Am J Clin Nutr*. 2001;74(2):188-196. doi:10.1093/ajcn/74.2.188

10. Xu Z, Niu L, Taylor JA. The ENmix DNA methylation analysis pipeline for Illumina BeadChip and comparisons with seven other preprocessing pipelines. *Clin Epigenetics*. 2021;13(1):216. doi:10.1186/s13148-021-01207-1

11. Xu Z, Niu L, Li L, Taylor JA. ENmix: a novel background correction method for Illumina HumanMethylation450 BeadChip. *Nucleic Acids Res*. 2016;44(3):e20. doi:10.1093/nar/gkv907

12. Niu L, Xu Z, Taylor JA. RCP: a novel probe design bias correction method for Illumina Methylation BeadChip. *Bioinformatics*. 2016;32(17):2659-2663. doi:10.1093/bioinformatics/btw285

13. Horvath S. DNA methylation age of human tissues and cell types. *Genome Biol*. 2013;14(10):3156. doi:10.1186/gb-2013-14-10-r115

14. Subar AF, Dodd KW, Guenther PM, et al. The food propensity questionnaire: concept, development, and validation for use as a covariate in a model to estimate usual food intake. *J Am Diet Assoc*. 2006;106(10):1556-1563. doi:10.1016/j.jada.2006.07.002

15. Mitry P, Wawro N, Six-Merker J, et al. Usual Dietary Intake Estimation Based on a Combination of Repeated 24-H Food Lists and a Food Frequency Questionnaire in the KORA FF4 Cross-Sectional Study. *Front Nutr*. 2019;6:145. doi:10.3389/fnut.2019.00145

16. Schwab S, Zierer A, Schneider A, et al. Vitamin E supplementation is associated with lower levels of C-reactive protein only in higher dosages and combined with other antioxidants: The Cooperative Health Research in the Region of Augsburg (KORA) F4 study. *Br J Nutr*. 2015;113(11):1782-1791. doi:10.1017/S0007114515000902

17. Lehne B, Drong AW, Loh M, et al. A coherent approach for analysis of the Illumina HumanMethylation450 BeadChip improves data quality and performance in epigenome-wide association studies. *Genome Biol*. 2015;16(1):37. doi:10.1186/s13059-015-0600-x

18. Pidsley R, Zotenko E, Peters TJ, et al. Critical evaluation of the Illumina MethylationEPIC BeadChip microarray for whole-genome DNA methylation profiling. *Genome Biol*. 2016;17(1):208. doi:10.1186/s13059-016-1066-1

19. McCartney DL, Walker RM, Morris SW, McIntosh AM, Porteous DJ, Evans KL. Identification of polymorphic and off-target probe binding sites on the Illumina Infinium MethylationEPIC BeadChip. *Genomics data*. 2016;9:22-24. doi:10.1016/j.gdata.2016.05.012

20. Houseman EA, Accomando WP, Koestler DC, et al. DNA methylation arrays as surrogate measures of cell mixture distribution. *BMC Bioinformatics*. 2012;13:86. doi:10.1186/1471-2105-13-86

21. Paalanen L, Männistö S, Virtanen MJ, et al. Validity of a food frequency questionnaire varied by age and body mass index. *J Clin Epidemiol*. 2006;59(9):994-1001. doi:10.1016/j.jclinepi.2006.01.002

22. Triche Jr TJ, Weisenberger DJ, Van Den Berg D, Laird PW, Siegmund KD. Low-level processing of Illumina Infinium DNA Methylation BeadArrays. *Nucleic Acids Res*. 2013;41(7):e90-e90. doi:10.1093/nar/gkt090

23. Baghurst KI, Record SJ. A computerised dietary analysis system for use with diet diaries or food frequency questionnaires. *Community Health Stud*. 1984;8(1):11-18. doi:10.1111/j.1753-6405.1984.tb00419.x

24. Ambrosini GL, de Klerk NH, O’Sullivan TA, Beilin LJ, Oddy WH. The reliability of a food frequency questionnaire for use among adolescents. *Eur J Clin Nutr*. 2009;63(10):1251-1259. doi:10.1038/ejcn.2009.44

25. Fortin JP, Fertig E, Hansen K. shinyMethyl: interactive quality control of Illumina 450k DNA methylation arrays in R. *F1000Research*. 2014;3:175. doi:10.12688/f1000research.4680.2

26. van Iterson M, Tobi EW, Slieker RC, et al. MethylAid: visual and interactive quality control of large Illumina 450k datasets. *Bioinformatics*. 2014;30(23):3435-3437. doi:10.1093/bioinformatics/btu566

27. Müller F, Scherer M, Assenov Y, et al. RnBeads 2.0: comprehensive analysis of DNA methylation data. *Genome Biol*. 2019;20(1):55. doi:10.1186/s13059-019-1664-9

28. Ainsworth BE, Haskell WL, Herrmann SD, et al. 2011 Compendium of Physical Activities. *Med Sci Sport Exerc*. 2011;43(8):1575-1581. doi:10.1249/MSS.0b013e31821ece12

29. Goldbohm RA, van den Brandt PA, Brants HA, et al. Validation of a dietary questionnaire used in a large-scale prospective cohort study on diet and cancer. *Eur J Clin Nutr*. 1994;48(4):253-265.

30. Feunekes GI, Van Staveren WA, De Vries JH, Burema J, Hautvast JG. Relative and biomarker-based validity of a food-frequency questionnaire estimating intake of fats and cholesterol. *Am J Clin Nutr*. 1993;58(4):489-496. doi:10.1093/ajcn/58.4.489

31. Baecke JA, Burema J, Frijters JE. A short questionnaire for the measurement of habitual physical activity in epidemiological studies. *Am J Clin Nutr*. 1982;36(5):936-942. doi:10.1093/ajcn/36.5.936

32. Graff M, Scott RA, Justice AE, et al. Genome-wide physical activity interactions in adiposity - A meta-analysis of 200,452 adults. *PLoS Genet*. 2017;13(4):e1006528. doi:10.1371/journal.pgen.1006528

33. Willett WC, Sampson L, Stampfer MJ, et al. Reproducibility and validity of a semiquantitative food frequency questionnaire. *Am J Epidemiol*. 1985;122(1):51-65. doi:10.1093/oxfordjournals.aje.a114086

34. Shimakawa T, Sorlie P, Carpenter MA, et al. Dietary intake patterns and sociodemographic factors in the atherosclerosis risk in communities study. ARIC Study Investigators. *Prev Med (Baltim)*. 1994;23(6):769-780. doi:10.1006/pmed.1994.1133
